# Pipelle Endometrial Biopsy for Abnormal Uterine Bleeding in Daily Clinical Practice: Why the Approach to Patients Should Be Personalized?

**DOI:** 10.3390/jpm11100970

**Published:** 2021-09-28

**Authors:** Naanlep M. Tanko, Faina Linkov, Gauri Bapayeva, Talshyn Ukybassova, Aiym Kaiyrlykyzy, Gulzhanat Aimagambetova, Kamila Kenbayeva, Bakytkali Ibrayimov, Alla Lyasova, Milan Terzic

**Affiliations:** 1 Clinical Academic Department of Laboratory Medicine, Pathology and Genetics, University Medical Center, Republican Diagnostic Center, 2 Syganaq Street, Nur-Sultan 010000, Kazakhstan; matthew.tanko@nu.edu.kz (N.M.T.); bakytkali.Ibrayimov@umc.org.kz (B.I.); 2 Department of Biomedical Sciences, School of Medicine, Nazarbayev University, Zhanybek-Kerey khans Street, 5/1, Nur-Sultan 010000, Kazakhstan; 3 Health Administration and Public Health Department, Rangos School of Health Sciences, Duquesne University, 600 Forbes Ave., Pittsburgh, PA 15282, USA; faina.linkov@gmail.com; 4 Department of Obstetrics, Gynecology and Reproductive Sciences, University of Pittsburgh School of Medicine, Pittsburgh, PA 15213, USA; 5 Clinical Academic Department of Women’s Health, National Research Center of Mother and Child Health, University Medical Center, Turan Ave. 32, Nur-Sultan 010000, Kazakhstan; gauri.bapayeva@gmail.com (G.B.); talshynu@yandex.ru (T.U.); camellia_enot@mail.ru (K.K.); milan.terzic@nu.edu.kz (M.T.); 6 National Laboratory of Astana, Nazarbayev University, Kabanbay Batyr Street, 53, Nur-Sultan 010000, Kazakhstan; aiym.kaiyrlykyzy@nu.edu.kz; 7 Pathology Bureau of Nur-Sultan city Administration, Zhansugirov Street, 1, Nur-Sultan 010000, Kazakhstan; lyasova.a@mail.ru; 8 Department of Medicine, School of Medicine, Nazarbayev University, Zhanybek-Kerey khans Street, 5/1, Nur-Sultan 010000, Kazakhstan

**Keywords:** Pipelle, dilation and curettage, endometrial sampling, endometrial carcinoma, endometrial hyperplasia, endometrial polyp, reliability, success rate, clinical practice

## Abstract

Background. Abnormal uterine bleeding (AUB) is a common gynecologic condition, and proper management is based on the histological evaluation of an adequate endometrial sample obtained via biopsy. The aims of this study were to evaluate factors influencing the reliability and success rate of Pipelle endometrial sampling for histopathological diagnosis. Methods. One hundred and eighty patients with AUB underwent endometrial sampling using both Pipelle and dilatation and curettage (D&C) procedures at the Clinical Academic Department of Women’s Health, University Medical Center between January 2019 and April 2021. We analyzed the effects of age, menopausal status, ethnicity, body mass index (BMI), provider experience, and procedure indication on the success and reliability of each procedure. Results. Pipelle sampling was successful in 144 (80.56%) women, while D&C was successful in 164 (91.11%) women. Analysis using Fisher’s exact test showed that age, menopausal status, and biopsy indication were factors affecting the success rate of both methods, while ethnicity, BMI, and physician experience had no influence. Overall concordance in the histopathological results between Pipelle and D&C was 91.72%. Conclusion. Pipelle sampling was found to be reliable for the detection of endometrial carcinoma and endometrial hyperplasia, while its reliability was low in cases of endometrial polyps. The endometrial sampling approach should be personalized in daily clinical practice for women with AUB, and Pipelle sampling is not suitable for all patients. If an endometrial polyp is suspected, the physician should consider other diagnostic tools, bearing in mind all of the factors influencing endometrial sampling success and reliability rates.

## 1. Introduction

Abnormal uterine bleeding (AUB) is one of the most frequent complaints in gynecologic patients. It affects 60–70% of women who are of reproductive and menopausal age [1]. In the reproductive age group, nearly 30% of outpatient clinic attendance is due to AUB [2]. Endometrial biopsy and histopathological evaluation can accurately diagnose the precursor lesions of endometrial carcinoma [3]. Although there are some attempts to utilize a transvaginal ultrasound scan for the detection of endometrial hyperplasia and even for differentiating benign hyperplasia from endometrial cancer at early stages, endometrial biopsy and histological evaluation remains the most reliable tool to diagnose the cause of abnormal uterine bleeding [4,5]. For nearly two decades, the Pipelle (aspiration) endometrial biopsy method has been used for the evaluation of AUB. Its use does not require general anesthesia or hospitalization and is performed as an outpatient or office procedure [2,3]. Some western countries have adopted the Pipelle endometrial sampling method as the preferred outpatient procedure in the evaluation of AUB due to the high accuracy in diagnosing endometrial cancer and its simplicity of use [6,7,8,9]. Despite these advantages of the Pipelle endometrial sampling method, to date, very few studies have been conducted to validate this method. Endometrial biopsy failure has been a major problem and has been reported by previously published studies. Clinical provider success rates with Pipelle procedures are well short of 100% (with a failure rate of up to a third) [10]. This is possibly due to multiple personal and medical factors: providers are unable to access the uterine cavity (e.g., due to patient discomfort, cervical stenosis, or inability to visualize the cervix), or there is an insufficient amount of tissue obtained for histological evaluation [11]. 

Endometrial cancer is the fifth most common cancer among women worldwide [12,13] and the third leading cancer in developed countries, accounting for 6–9% of all cancers in women [12]. The incidence of endometrial cancer in Asia varies among countries and was reported to be the highest in Armenia (26.7 per 100,000 population) followed by Israel (15.4 per 100,000 population) in the age group of women aged 60–64-years-old, while the highest mortality rate due to this cancer is in Armenia (1.6 per 100,000 population) followed by Afghanistan (1.4 per 100,000 population) [12]. According to the available data, in Kazakhstan, a Central Asian post-Soviet republic, there were 1259 new endometrial cancer cases reported in 2012 (incidence rate 5.8%), with 280 patients dying due to the disease [12,13].

Considering the importance of AUB in clinical practice as well as the high rate of en-dometrial cancer in Kazakhstan, the need to validate the Pipelle endometrial sampling in clinical settings was found to be necessary. Therefore, the overall goal of our research was to fill important gaps in the current understanding of Pipelle endometrial biopsy feasibility in the country. The first specific aim was to explore the factors influencing the Pipelle sampling success rate, while the second aim was to evaluate the histopathological diagnostic accuracy of Pipelle endometrial sampling.

## 2. Methods

### 2.1. Setting and Study Participants

This prospective cross-sectional study evaluated the factors influencing the Pipelle endometrial sampling success rate and the accuracy of the histopathological diagnosis on the endometrial tissues obtained via Pipelle endometrial biopsy and dilatation and curettage in women with abnormal pre- and postmenopausal uterine bleeding. All of the study participants were recruited at the Clinical Academic Department of Women’s Health of the University Medical Center (UMC), Nur-Sultan City, the Republic of Kazakhstan. The study protocol was approved by the Institutional Research Ethics Committee of the Nazarbayev University (NU IREC) and the University Medical Center Institutional Research Board (UMC IRB), (25 February 2019, number: 109/11122018). Each study participant gave written informed consent. Participants were recruited into the study if there was indication for D&C and if they met the inclusion criteria: (1) age ≥ 18 years, (2) intact uterus or cervix, and (3) abnormal uterine bleeding or irregular cycles (if there were premenopausal) or postmenopausal bleeding. Women with any of the following conditions were excluded from the study: (1) age < 18 years, (2) comorbid conditions such as cervical cancer, pregnancy, acute pelvic inflammatory disease, acute cervicitis or vaginitis, clotting disorders, uterine malformations, previous hysterectomy, previous uterine ablation, and having undergone previous procedures for Asherman’s syndrome.

### 2.2. Survey Data

After a thorough explanation of the project aims, the procedure, and the required investigations, 180 consecutive participants signed the informed consent form. The baseline clinical and socio-demographic data of the participants were obtained through questionnaires and patient medical records. A provider questionnaire was developed to record the indication for the procedure, analgesic use, biopsy success or failure, reason for failure, and patient ultrasound record. Biopsy failure was defined as the inability to access the endometrium or the inability to obtain adequate endometrial tissue for histological examination. 

### 2.3. Endometrial Sample Collection

Pipelle endometrial sampling was conducted in the gynecological outpatient clinic of the UMC. If the tissue obtained was considered inadequate under visual assessment, the procedure was repeated to optimize sampling. The endometrial tissues obtained were fixed in 10% buffered formalin and were transported to the pathology laboratory for histopathological studies. The patient was then transferred to the operating room for D&C under general anesthesia. The D&C was performed according to hospital protocols, and the endometrial tissues were fixed in 10% buffered formalin, as described above. The procedures were performed by a senior (>35 years of experience) and a junior (<5 years of experience) specialist in obstetrics and gynecology (OBGYN). The coupled endometrial biopsy samples were subjected to histopathological studies. Histopathological evaluation and diagnosis included all of the morphologic abnormalities that were observed in the coupled samples. 

### 2.4. Statistical Analysis

We used descriptive statistics to analyze the sociodemographic and clinical parameters of the patients, as well the histopathological diagnosis on both the Pipelle and D&C samples. Continuous variables were described as the median and interquartile range. The Wilcoxon rank-sum test (continuous variables) and Fisher’s exact test (categorical variables) were used to compare women’s ages, menopausal status, ethnicity, type of healthcare provider, and indication for current biopsy. *p*-values < 0.05 were considered significant. A 2-by-2 table was used to calculate the sensitivity (SN), specificity (SP), positive predictive value (PPV), and negative predictive value (NPV) of both the Pipelle versus the D&C samples. The accuracy of the test is the overall probability that a test correctly diagnoses or classifies the pathologic entity. The SN, SP, PPV, NPV, disease prevalence, and diagnostic accuracy are expressed as percentages. The data were analyzed using Stata version 13. 

## 3. Results 

The study population consisted of 180 patients who underwent a coupled (Pipelle and D&C) endometrial sampling from January 2019 to April 2021 at the Clinical Academic Department of Women’s Health of the UMC, Nur-Sultan City, Kazakhstan. The characteristics of the study population is represented descriptively in Table 1. Based on age, all of the patients (180) in the study were divided into three age groups: (1) ≤44 years of age, (2) 45–54 years of age, and (3) ≥55 years old (Table 1).

The success rate of the biopsy performed with the Pipelle device was the highest in the group of patients who were ≤44 years of age followed by those who were 45–54 years of age (93.33% and 78%, respectively). In older patients (≥55 years-old), the success of this method was much lower, at 32%. The differences observed between all three age groups were statistically significant (*p* < 0.001). When these patients were sorted according to the menopausal state, success rate was higher in the premenopausal group than it was in postmenopausal group (Table 1), (*p* < 0.001). 

In the D&C endometrial sampling, the success of biopsy was the highest in the group of women who was ≤ 44 years of age followed by the group of women who were 45–54 years of age (Table 1). In patients older than 55, the success of D&C sampling was 68%. These differences between the three age groups were statistically significant (*p* < 0.001). 

In terms of ethnicity, the majority of the patients (80.56%) were Asian (predominantly of Kazakh origin) (Table 1). However, there was no significant differences in terms of the success rate of Pipelle and D&C based on the ethnicity (*p* = 0.570 and *p* = 0.317, respectively).

The association between BMI and Pipelle success rate was analyzed as well and is presented in Table 1. In the Pipelle group, adequate material for pathology diagnostics was obtained in 86.84% of the patients with normal BMI. In the group of overweight and obese women, the success rate was 75.96%. The difference between groups was not statistically significant (*p* = 0.068).

In the D&C group, adequate material for pathology diagnostics was obtained in 92.11% of patients with normal BMI and in 90.38% of overweight and obese women. The difference between groups was not statistically significant (*p* = 0.689).

As Pipelle biopsy sampling failed in 35 out of 180 (19.44%) women, we analyzed physician experience on specimen adequacy. The senior OBGYN provider was unsuccessful in 20 out of 115 (17.39%) patients compared to the 15 out 65 (23.08%) failure rate of a junior OBGYN specialist (Table 1). Around 43% of failures occurred due to the junior physician—15 out of 35), but this difference was not statistically significant (*p* = 0.335).

From the other side, D&C endometrial sampling failed in 16 out of 180 (8.89%) women. Physician experience did not have a statistically significant influence on specimen adequacy: the senior OBGYN provider was unsuccessful in 12 out of 103 (10.43%) patients, while the junior physician failed in 4 out 61 (6.15%) patients (*p* = 0.420, Table 1). 

The indication for the biopsy was another parameter that was analyzed. Diagnostic biopsies were most commonly performed in patients with abnormal uterine bleeding in reproductive age 60%, followed by premenopausal and postmenopausal bleeding: 22.78% and 17.22%, respectively (Table 1). Pipelle success rates were significantly different depending on the indication for endometrial sampling (*p* < 0.001), being successful in 93.52% of patients of reproductive age experiencing bleeding and 80.49% and 35.48% successful in patients of pre- and postmenopausal age experiencing bleeding, respectively. 

In the D&C, group diagnostic biopsies were mostly successful in patients with abnormal bleeding in reproductive age (97.22%) followed by premenopausal and postmenopausal patients experiencing bleeding as a sampling indication at 90.24% and 70.97%, respectively (Table 1). D&C success rates were significantly different depending on the indication for endometrial sampling, being the lowest in postmenopausal bleeding patients (*p* < 0.001).

**Figure 1:**
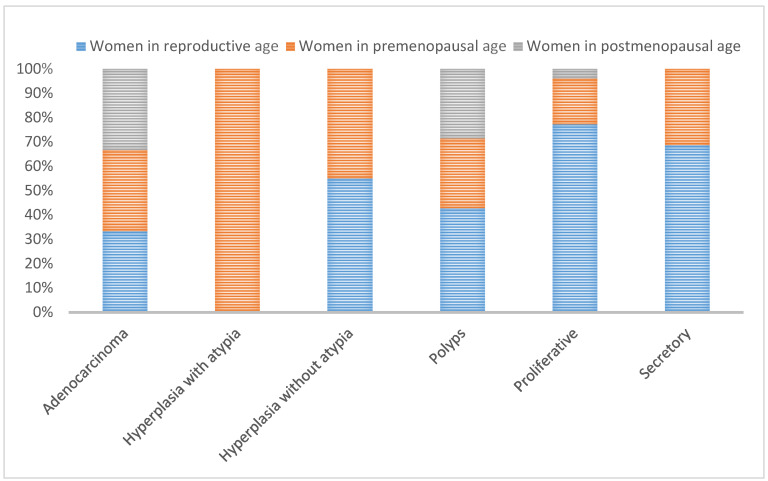
Results of histopathological diagnosis of endometrial samples by age group (Pipelle biopsy).

**Figure 2:**
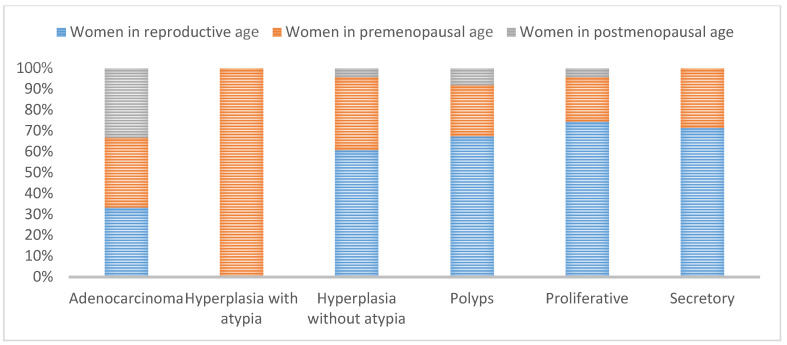
Results of histopathological diagnosis of endometrial samples by age group (D&C).

**Figure 3:**
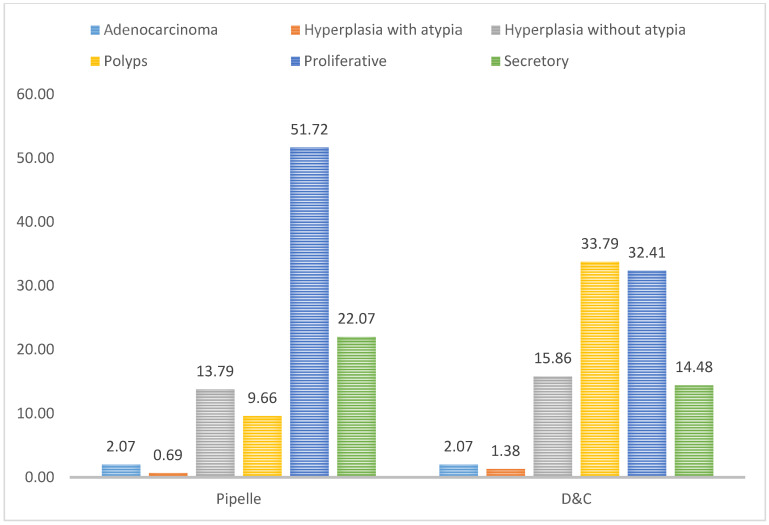
Comparison of histopathological diagnosis of Pipelle and D&C endometrial biopsy samples.

The endometrial tissues obtained for histopathology was 91.11% adequate when the procedure was D&C, while tissue adequacy was 80.56% in Pipelle biopsy samples (Figure 1 and Figure 2). Figure 3 shows a graphic comparison of the histopathological diagnosis of the endometrial samples obtained via both Pipelle and D&C methods.

The final analysis with adequate samples included 145 patients. The overall concordance between the Pipelle and D&C histopathological diagnosis was 91.72% (Table 2). The most common histopathological diagnosis for both the Pipelle and D&C samples was proliferative endometrium. Table 3 shows the diagnostic reliability of the Pipelle technique in identifying different endometrial pathologies, ranging in accuracy from 90.97% to 100%. 

Pipelle sampling was found to have a sensitivity, specificity, PPV, NPV, and accuracy of 100% for the diagnosis of endometrial carcinoma and 72% sensitivity, 97.48% specificity, a PPV of 85.71%, a NPV of 94.31%, and accuracy of 93.06% for endometrial hyperplasia. Of the three cases of adenocarcinoma that were diagnosed by both methods, one was in a postmenopausal woman, and two were in premenopausal women. 

A total of 49 out of the 145 patients had a pathological diagnosis of endometrial polyps on the D&C samples (33.8%). However, endometrial polyps were only diagnosed in 11 (22.45%) of these 49 cases on the Pipelle samples. This may be due to sampling error by the Pipelle because of the focal nature of endometrial polyps. Overall, the PPV of the Pipelle endometrial samples in detecting endometrial polyps was 78.57%. This PPV was 83.33% in reproductive age women, 75% in premenopausal women, and 75% in postmenopausal women. The overall accuracy rate of the Pipelle for the detection of endometrial polyps was 71.53% (Table 4). 

## 4. Discussion

Regular monthly uterine bleeding remains an integral part of overall women’s health [14]. Abnormal uterine bleeding (AUB) is frequent in daily practice, and in order to simplify management and enhance the well-being of women, physicians need to follow current guidelines and recommendations [15,16]. From the other side, both the adequacy of endometrial tissue obtained during the procedure for histological analysis and the endometrial sampling success rate is influenced by many factors in daily practice. 

Regarding the pathogenesis of endometrial pathologies, various multiple genetic (non-modifiable) and non-genetic (modifiable) risk factors have been associated with the development of different endometrial entities including, endometrial cancer [17,18,19,20]. 

This is the first study assessing the feasibility of Pipelle endometrial sampling in Kazakhstan, with the results probably having an impact to Governmental policy definitions and approaches for patients with abnormal uterine bleeding. With the collapse of the Soviet Union in 1991, Kazakhstan has experienced economic recession, and the healthcare system of the country has gone through three decades of restructuring [21]. Despite recent improvements, the country still lags behind other post-soviet independent states of the European Region on key indicators of health and economic development [22]. 

Kazakhstan has higher rates of mortality from endometrial malignancies compared to developed western countries, possibly due to lack of timely diagnosis [23]. Pipelle biopsies are not practiced in the governmentally sponsored healthcare facilities and are only available in some private clinics. The introduction of Pipelle endometrial sampling in ambulatory care settings is needed to improve the rate of early diagnosis of endometrial pathologies, to help curb the increasing costs of gynecologic care, and to improve overall patient outcomes. As of April 2020, Pipelle biopsy is not commonly used in Kazakhstan, and most endometrial tissue assessments are conducted using conventional (blind) D&C, typically performed as an inpatient procedure in the operating rooms. In 2018, the Ministry of Healthcare of the Republic of Kazakhstan approved the guidelines for the management of endometrial hyperplastic disorders and suggested the utilization of Pipelle biopsy for monitoring endometrial histology during hormone therapy but not for screening examination [24,25].

In our study, the success rate for endometrial biopsy sampling varied according to many patient variables. The overall success rate in obtaining adequate endometrial tissue was 80.56% for the Pipelle method and 91.11% for D&C. We found that younger age group, premenopausal bleeding, and AUB in reproductive age were significant predictors of success (*p* < 0.001). Ethnicity, provider experience, and BMI did not affect the success rate of the Pipelle endometrial sampling. Our findings of high failure rates in older age group and postmenopausal bleeding in agreement with the previous studies [11,26,27]. The reason for the high failure rate in the different categories of patients may be due to postmenopausal atrophy of the endometrial tissue and endometrial cavity obliteration or narrowing, thereby making endometrial tissue less available for sampling. 

We found that adenocarcinoma diagnosis was 100% reliable in all the statistical variables. These findings show that the Pipelle has a low sensitivity despite being highly specific. The low sensitivity might be related to missing polyps as endometrial focal lesions. However, if endometrial pathology was present and sampled by the Pipelle, histopathological diagnosis was accurate, with specificity of 100%, 97.52%, and 100% for hyperplasia with atypia, hyperplasia without atypia, and adenocarcinoma, respectively. The results of our study are comparable with several previous reports. Sarwar et al. found that the Pipelle had a sensitivity of 100%, a specificity of 98%, and 100% NPV when detecting endometrial hyperplasia with and without atypia. Their higher sensitivity value could be due the fact that focal endometrial lesions were rare in their sample [28]. In another study, the Pipelle had a SN, SP, PPV, NPV, and accuracy of 64.2%, 88.8%, 94.1%, 85.5%, and 47.3% for hyperplasia, respectively [29]. They concluded that the low sensitivity and accuracy might have been due to the over-detection of secretory and proliferative endometrium as hyperplasia. Recent investigation of the diagnostic accuracy of the Pipelle aspiration biopsy and dilatation and curettage (D&C) in patients diagnosed with endometrial hyperplasia prior to hysterectomy found that D&C more accurately reflected the final diagnosis in patients with endometrial hyperplasia than aspiration biopsy based on the histological examination of hysterectomy specimens [30]. Regarding sampling adequacy and sensitivity, another recently published study confirmed that Pipelle performed as well as dilation and curettage and even better than other endometrial sampling devices [31]. 

In our study, 99.31% and 93.75% of endometrial hyperplasia with and without atypia were accurately diagnosed using Pipelle. In contrast, the sensitivity of the Pipelle in detecting endometrial polyps was low, at 22.45%. In previous studies, the Pipelle was shown to be weak in diagnosising endometrial polyps and other focal lesions. Vinita et al. found that out of eight polyps that were diagnosed on hysteroscopy-guided biopsy samples in women with AUB, only one (12.5%) was diagnosed using Pipelle [2]. The accuracy of the Pipelle in detecting endometrial polyps was a dismal 16%. In another previous study [32], the accuracy of the Pipelle in detecting endometrial polyps was 16%, while Ilavarasi and coauthors (2019) were not able at all to detect endometrial polyps using the Pipelle [29]. In a recently published evidence-based diagnosis and management guide of postmenopausal women with vaginal bleeding and suspected endometrial polyp on ultrasound scans, diagnostic hysteroscopy with hysteroscopic polypectomy was suggested [33]. Accordingly, the approach to patients with AUB in daily clinical practice should be personalized, considering patient’s age, history data, clinical complaints/presentation, and eventual availability of vaginal ultrasound scan before the procedure. 

Strengths and limitations. Compared to previous studies that attempted to compare the diagnostic accuracy of the Pipelle and D&C endometrial biopsy methods, our study has several strengths. First, we had a substantial number of participants (180). Second, this was the first study assessing the feasibility of the Pipelle in Kazakhstan, with great implications for future policy definitions. Third, we the excluded impact of physician clinical experience length on the Pipelle success rate, thus enabling this tool to be available and advisable for all trained doctors. Fourth, we excluded patients who had any conditions that could be a confounding factor such as hormonal therapy and comorbid entities such as cervical cancer, pregnancy, acute pelvic inflammatory disease, acute cervicitis or vaginitis, clotting disorders, uterine malformations, previous hysterectomy, previous uterine ablation, and previous procedures completed for Asherman’s syndrome. Finally, this study assesses the factors influencing the failure rate in a very important clinical setting servicing patients from various regions of Kazakhstan. 

The only limitation of the study is the lack of follow up of women in which both methods were unsuccessful in obtaining endometrial tissue. 

In conclusion, adequate samples for histological evaluation were obtained using the Pipelle in 80.56% and using D&C in 91.11% of patients. Failure to obtain an adequate specimen for histological analysis is possible following both the Pipelle and D&C methods. The indications for endometrial biopsy and patient age were factors affecting both the Pipelle and D&C success rates. Physician experience, BMI, and patient ethnicity were not statistically relevant for the success rate of either the Pipelle or D&C. Pipelle biopsy was highly reliable for the diagnosis of endometrial hyperplasia and endometrial carcinoma. Based on the results of our study, we strongly recommend that the endometrial sampling approach should not be the same for all patients with abnormal uterine bleeding. Each case should be assessed individually, and decisions related to sampling procedures and devices should be personalized and customized, considering all of the factors influencing both the success and reliability rate. However, since the Pipelle biopsy is a cheap, simple to handle, save, well tolerated, and a reliable office or outpatient tool, we recommend that be the initial diagnostic method in the evaluation of AUB, except for in patients with ultrasound scan results showing focal lesions such as endometrial polyps. The more expensive procedures in the operating room should be reserved for selected patients who are not good candidates for Pipelle.

## Figures and Tables

**Table 1:** Pipelle endometrial sampling failure rate: impact of age, BMI, ethnicity, indication, and provider type *.

Variables	N (%)	Pipelle Sampling		*p*	D&C Sampling		*p*
		Failure Rate N = 35, 19.44%	Success Rate N = 145, 80.56%		Failure Rate N = 16, 8.89%	Success Rate N = 164, 91.11%	
**Age group (N = 180)**				<0.001			<0.001 *
≤44	105 (58.33)	7 (6.67)	98 (93.33)		3 (2.86)	102 (97.14)	
45–54	50 (27.78)	11 (22)	39 (78)		5 (10)	45 (90)	
≥55	25 (13.89)	17 (68)	8 (32)		8 (32)	17 (68)	
**Menopausal status (N = 180)**				<0.001			<0.001 *
Premenopausal women	144 (80)	13 (9.03)	131 (90.97)		5 (3.47)	139 (96.53)	
Postmenopausal women	360(20)	22 (61.11)	14 (38.89)		11 (30.56)	25 (69.44)	
**Ethnicity (N = 180)**				0.570			0.317 *
Asian (Kazakh, Tatar etc)	145 (80.56)	27 (18.62)	118 (81.38)		15 (10.34)	130 (89.66)	
Non-Asian (Russian, Ukrainian, German etc)	35 (19.44)	8 (22.86)	27 (77.14)		1 (2.86)	34 (97.14)	
**BMI group (N = 180)**				0.068			0.689
Normal (≤24.9)	76 (42.22)	10 (13.16)	66 (86.84)		6 (7.89)	70 (92.11)	
Overweight and obese(≥25.0)	104 (57.78)	25 (24.04)	79 (75.96)		10 (9.62)	94 (90.38)	
**Type of provider (N = 180)**				0.335			0.420 *
Senior OBGYN specialist	115 (63.89)	20 (17.39)	95 (82.61)		12 (10.43)	103 (89.57)	
Junior OBGYN specialist	65 (36.11)	15 (23.08)	50 (76.92)		4 (6.15)	61 (93.85)	
**Indications (N = 180)**				<0.001			<0.001 *
Abnormal bleeding in reproductive age	108 (60)	7 (6.48)	101 (93.52)		3 (2.78)	105 (97.22)	
Premenopausal bleeding	41 (22.78)	8 (19.51)	33 (80.49)		4 (9.76)	37 (90.24)	
Postmenopausal bleeding	31 (17.22)	20 (64.52)	11 (35.48)		9 (29.03)	22 (70.97)	

* Fisher’s exact test was used to test the differences in groups. Abbreviation: OBGYN—obstetrics and gynecology.

**Table 2:** Diagnostic concordance of histopathological studies of D&C and Pipelle endometrial samples.

Endometrial Histopathology Report	Endometrial Histopathology on Pipelle, N	Endometrial Histopathology on D&C, N	Concordance in Histopathological Diagnosis, %
Adenocarcinoma	3	3	100
Hyperplasia	21	23	91.3
Proliferative	89	84	94.3
Secretory	32	33	96.9
Total	145	145	91.72

**Table 3:** Analysis of overall Pipelle biopsy reliability.

Endometrial Characteristics	Sensitivity, % (95% CI)	Specificity, % (95% CI)	Positive Predictive Value, % (95% CI)	Negative Predictive Value, % (95% CI)	Accuracy, % (95% CI)
Hyperplasia, including:	72.00 (50.61–87.93%)	97.48 (92.81–99.48%)	85.71 (65.66–94.96%)	94.31 (89.83–96.88%)	93.06 (87.60–96.62%)
Hyperplasia with atypia	50.00 (1.26–98.74%)	100.00 (97.44–100.00%)	100.00	99.30 (97.26–99.82%)	99.31 (96.19–99.98%)
Hyperplasia without atypia	73.91 (51.59–89.77%)	97.52 (92.93–99.49%	85.00 (64.36–94.68%	95.16 (90.81–97.51%)	93.75 (88.47–97.10%)
Adenocarcinoma	100 (2.50–100.00%)	100 (97.45–100.00%)	100	100	100 (97.47–100.00%)
Proliferative	95.24 (88.25–98.69%)	85.00 (73.43–92.90%)	89.89 (82.93–94.21%)	92.73 (82.97–97.09%)	90.97 (85.06–95.11%
Secretory	93.94 (79.77–99.26%)	99.11 (95.13–99.98%)	96.88 (81.47–99.54%)	98.23 (93.55–99.53%)	97.93 (94.07–99.57%)

**Table 4:** Reliability of Pipelle biopsy for the evaluation of endometrial polyps.

Variables	Overall	Reproductive Age	Premenopausal Age	Postmenopausal Age
Sensitivity, % (95% CI)	22.45 (11.77–36.62%)	15.15 (5.11–31.90%)	25.00(5.49–57.19%)	75 (19.41–99.37%)
Specificity, % (95% CI)	96.84 (91.05–99.34%)	98.46 (91.72–99.96%)	96.15 (80.36–99.90%)	75 (19.41–99.37%)
Positive predictive value, % (95% CI)	78.57 (51.75–92.61%)	83.33(37.84–97.62%)	75.00 (25.75–96.29%)	75 (33.39–94.72%)
Negative predictive value, % (95% CI)	70.77 (67.46–73.87%)	69.57 (66.36–72.59%)	73.53 (66.51–79.53%)	75 (33.39–94.72%)
Accuracy, % (95% CI)	71.53 (63.42–78.73%)	70.41 (60.34–79.21%)	73.68 (56.90–86.60%)	75 (34.91–96.81%)

## Data Availability

All data related to this study are available from the project PI, Professor Milan Terzic; email: milan.terzic@nu.edu.kz.
